# *Ginkgo biloba* Extract Inhibits Astrocytic Lipocalin-2 Expression and Alleviates Neuroinflammatory Injury via the JAK2/STAT3 Pathway After Ischemic Brain Stroke

**DOI:** 10.3389/fphar.2018.00518

**Published:** 2018-05-16

**Authors:** Yehao Zhang, Jianxun Liu, Bin Yang, Yongqiu Zheng, Mingjiang Yao, Mingqian Sun, Li Xu, Chengren Lin, Dennis Chang, Fangze Tian

**Affiliations:** ^1^Beijing Key Laboratory of Pharmacology of Chinese Materia Region, Institute of Basic Medical Sciences of Xiyuan Hospital, China Academy of Chinese Medical Sciences, Beijing, China; ^2^National Institute of Complementary Medicine, Western Sydney University, Penrith, NSW, Australia

**Keywords:** cerebral ischemia, neuroinflammation, astrocyte, Lipocalin-2 (LCN2), *Ginkgo biloba* extract

## Abstract

**Background:** Astrogliosis has the potential to lead to harmful effects, namely, neuroinflammation, and to interfere with synapse sprouting. Previous studies have suggested that Lipocalin-2 (LCN2) acts as a key target in regulating the reaction of astrocytes. However, the underlying molecular mechanism is not fully elucidated. In the present study, we examined the neuroprotective and anti-inflammatory effects of *Ginkgo biloba* extract (EGB), a well-known extract with potential immunoregulatory properties in the nervous system.

**Methods:** Triphenyltetrazolium chloride staining, hematoxylin-eosin staining, electron microscopy, and neurological assessments were performed in a microsphere-embolized rat model. Human astrocytes exposed to oxygen glucose deprivation (OGD) were used for *in vitro* experiments. Inflammatory cytokines, multi-labeling immunofluorescence, and Western blotting were used to investigate the molecular mechanisms underlying the EGB-mediated anti-inflammatory effects *in vivo* and *in vitro*.

**Results:** EGB markedly attenuated cerebral infarction and neuronal apoptosis, reduced the inflammatory cytokine level, and alleviated neurological deficiencies in cerebral ischemic rats. After surgery, EGB significantly inhibited astrocyte activation, reduced the phosphorylation of STAT3 and JAK2 and decreased LCN2 expression. *In vitro*, EGB blocked OGD-induced STAT3 activation and the generation of pro-inflammatory cytokines in human astrocytes, and these effects were significantly enhanced by LCN2 overexpression. EGB downregulated these effects enhanced by LCN2 overexpression.

**Conclusion:** EGB is demonstrated to mediate neuroinflammation, which protects against ischemic brain injury by inhibiting astrogliosis and suppresses neuroinflammation via the LCN2-JAK2/STAT3 pathway, providing insight into a promising therapeutic strategy for ischemic stroke.

## Introduction

Stroke is the primary reason for mortality worldwide, is responsible for approximately 6 million deaths annually and is considered the key reason for long-term disability (Mendis et al., 2015). Acute cerebral ischemia is caused in the great majority of stroke cases by the occlusion of a supplying arterial vessel (Mozaffarian et al., 2016). Inflammation and immune responses are considered essential for the development of strokes (Fu et al., 2015). The immune system is closely related to crucial events determining the fate of the ischemic brain and the survival of stroke patients. Inflammation may ultimately lead to neuronal cell death by inducing the entry of leukocytes (Lucas et al., 2006), the release of inflammatory cytokines (Lambertsen et al., 2012), and apoptosis (Pettigrew et al., 2008). Some reagents that ameliorate ischemia-induced inflammation have a protective effect, (Zhang et al., 1994; Furuya et al., 2001) suggesting that inflammation may be the crucial mechanism responsible for ischemic brain injury (Chamorro and Hallenbeck, 2006).

Astrocytes respond to attacks on the CNS through a process called astrogliosis (Barres, 2008). Several studies have clarified the capabilities and effects of astrogliosis. Through the release of diverse molecules, reactive astrocytes can have several impacts on nearby cells (Pekny and Nilsson, 2005; Sofroniew, 2009). For example, astrocytes can produce a broad repertoire of pro-inflammatory and anti-inflammatory cytokines to target tissue damage following trauma, ischemia, infection, degenerative disease, or autoimmune inflammation (Argaw et al., 2012; Zamanian et al., 2012; Kim et al., 2014). Recently, studies have demonstrated that LCN2 is related to a number of CNS injury conditions, such as cerebral ischemia (Jin et al., 2014), excitotoxic injury, stab wound (Chan et al., 2014), medial forebrain bundle transection, and lipopolysaccharide (LPS)-induced neuroinflammation (Suk, 2016). Reactive astrogliosis is involved in the pathogenic mechanisms of these disease, and LCN2 expression is induced in reactive astrocytes (Jin et al., 2014). Therefore, astrocytes and LCN2 may be potential molecular therapeutic targets for clinicians to treat inflammatory CNS injuries.

The therapeutic actions of *Ginkgo biloba* have been well-known in traditional Chinese medicine for over 5000 years. In 1965, *G. biloba* leaf extract was first introduced and registered for use in medical practice (DeFeudis, 2003), which commonly used for ailments, including peripheral arterial disease, Alzheimer’s disease, short-term memory loss, depression, and anxiety (Kleijnen and Knipschild, 1992; Curtis-Prior et al., 1999). Accordingly, several of these molecular mechanisms have been analyzed in various stroke models. Several studies have demonstrated that EGB has a wide range of therapeutic activities, such as mitochondrial function recovery (Baliutyte et al., 2014), antioxidation (Mohamed and Abd El-Moneim, 2017), anti-inflammation (Li et al., 2017), and apoptosis inhibition (Wang et al., 2015).

In the present study, we investigated the extent to which EGB can alleviate reactive astrocytes and mediate the repair phase in stroke. We used *in vitro* and *in vivo* cerebral ischemia models to investigate the extent that EGB can suppress neuroinflammation, and we investigated possible associated mechanisms. Our results showed that EGB significantly inhibited cerebral ischemia development by downregulating the LCN2-activated JAK2/STAT3 signaling pathway and inhibiting astrocyte proliferation. Therefore, our findings indicated a novel role for EGB in the treatment of cerebral ischemia.

## Materials and Methods

### Microsphere-Embolized Rat Model

We used male SD (Sprague Dawley) rats with a weight of 220–250 g (Beijing Vital River Laboratory Animal Technology Co., Ltd., Beijing, China). All the procedures and ethics guidelines were approved by the Committee for Experimental Animal Use and Care of the Chinese Academy of Medical Sciences, China. Efforts were made to minimize the number of animals used and their suffering. The rats were housed at a temperature and humidity of 23 ± 1°C and 55 ± 5%, respectively, with a 12-h light-dark cycle. Rats had unrestricted access to food and water.

All 50 adult SD rats were randomly allocated into five groups. Microsphere-induced cerebral embolism was conducted with a previously described method (Moriyama et al., 2013). After anesthetization with 40 mg/kg chloral hydrate, the common carotid artery and right external carotid of the rats were temporarily occluded with vascular clamps (*n* = 10 per group, 5 groups). A 2-mL syringe was then inserted into the right internal carotid. Microspheres (106–212 μm in diameter, UVPMS-BY2, Cospheric, United States) were suspended in rat serum with 1 mg/ml, and 0.2 mL of this suspension was injected into the right internal carotid artery. After the injection, the vascular clamps occluding the right external and common carotid arteries were released, and then, the puncture wound was closed with suture strings. Blood flow to the brain by the right external and common carotid arteries recommenced after 2–3 s. Rats in the sham group were injected with the same volume of rat serum but without microspheres.

### Drug Administration

*Ginkgo biloba* extract were provided in-kind by Shineway Pharmaceutical Group (Shijiazhuang, China), it contains total ginkgo flavone-glycosides from *G. biloba*. The quantitative analysis results showed that it contained 49% of total flavones (UV), 28.7% of sum of glycosides with aglycone of quercetin, isorhamnetin and kaempferol (HPLC-UV), 11.6% of sum of gingkolide A, B, C and bilobalide with 3.3% of gingkolide A (HPLC-UV) among them in gingko extract (Zhang et al., 2018). The EGB preparations were manufactured in a Good Manufacturing Practice certified facility. EGB was injected into the duodenum of the rats after cerebral ischemia surgery at doses of 7.5 and 15 mg/kg. Then EGB was intragastrically administrated at 24 h and 48 h after surgery. Rats in the sham group were administered the same volume of saline.

### Evaluation of Neurological Deficits

Scores pertaining to the neurological deficits of the rats were calculated for each group after 2 h of ischemia and 24 h. These scores were conducted based on a five-point scale adapted from a previous publication (Bederson et al., 1986). Specifically, if a rat demonstrated normal spontaneous movements (no neurological deficit), they were assigned a score of 0; if a rat was unable to fully extend its right paw, they were assigned a score of 1; if a rat circled in a clockwise motion, they were assigned a score of 2; if a rat fell to the right, they were assigned a score of 3; if a rat was unable to walk, they were assigned a score of 4. All neurological assessments were conducted by a researcher blinded to all groups.

### Measurement of Cerebral Infarction

Forty-eight hours after cerebral ischemia, 40 mg/kg chloral hydrate was administered to anesthetize the rats. Before decapitation, blood serum was collected using the abdominal aorta method. The brain was then detached, and 1-mm-thick coronal slices were acquired and fixed in prewarmed 2 % TTC for 10 min. Finally, the slices were fixed in 10% paraformaldehyde for 30 min.

### Electron Microscopy

At 48 h after cerebral ischemia, cerebral cortex slices measuring 1 cubic millimeter were crafted into square pieces with a vertex at the center of the ischemic area comprising part of the penumbra and undamaged area. These pieces were fixed for 2 h with 2.5% glutaraldehyde in PBS at room temperature. The pieces were washed with PBS, incubated for 1 h in PBS solution containing 1% OsO_4_, dehydrated with ethanol, contrast-stained with 1% uranyl acetate, and embedded in EPON resin. Ultrathin sections were prepped and observed using the ultramicrotome Leica EM UC6 and a transmission electron microscope HITACHI H-7500 (HITACHI, Japan), respectively.

### Human Astrocyte Culture and Transfection

Astrocytes were purchased from ScienCell Research Laboratories (CA, United States). In this study, cells were grown in Normal astrocyte media (AM) (Sicencell, United States) containing 10% FBS (Gibco, United States), 1% l-glutamine, and 1% penicillin-streptomycin in a humidified atmosphere of 5% CO_2_ at 37°C. The expression vector of the LCN2 gene (EX-m0282-Lv201) and the vector control (EX-NEG-Lv201) were obtained from GeneCopoeia (Rockville, United States). After cells reached 70% confluence, the astrocytes were stably transfected using 7.5 μl/ml lentiviral vector (LPP-m0282-Lv201-100) from GeneCopoeia (Rockville, United States) for 24 h. Then, the cells were given normal astrocyte media (AM) (Sicencell, United States) containing 10% FBS (Gibco, United States) without lentiviral vector for 48 h.

### Oxygen Glucose Deprivation (OGD) Management and Treatment

After purification and transfection, human astrocytes were cultured in an incubator with premixed gas (1% O_2_, 94% N_2_, 5% CO_2_) for 6 h using deoxygenated DMEM without glucose or FBS. After 6 h, the cells were given normal AM containing 10% FBS and were transferred to a CO_2_ incubator with 95% air and 5% CO_2_ environment for 12 h. The cells in the control group were simultaneously cultured with normal AM and 10% FBS. During OGD induction, astrocytes were treated by 2.5, 5, or 10 mg/L of EGB for 6 h.

### Western Blotting

The protein from tissues containing ischemic penumbra were harvested at 24, 48 h after cerebral ischemia was extracted by RIPA buffer (Beyotime Biotechnology, Shanghai, China) and was mixed with protease and phosphatase inhibitor cocktails (MCE, NJ, United States). The protein concentration was determined using a protein assay solution (Bio-Rad). Identical quantities of protein were denatured using protein loading buffer, loaded onto 10% SDS–PAGE gels, and transferred to polyvinylidene difluoride (PVDF) membranes by electroblotting. The PVDF membranes were blocked by 5% bovine serum albumin (BSA) in TBST buffer for 1 h and were incubated overnight at 4°C using the following antibodies: LCN2 (Abcam, 1:2000 dilution), GFAP (Proteintech, 1:2000), p-JAK2 (Tyr1007/1008) (Abcam, 1:1000), JAK2 (Abcam, 1:2000), p-STAT3 (Tyr705) (Cell Signaling Technology, 1:2000), STAT3 (Cell Signaling Technology, 1:2000), and β-actin (Sigma, 1:5000). Reactive bands were detected using ECL detection reagent (Thermo Fisher Scientific, MA, United States) following the manufacturer’s instructions. The protocols for cell culture experiments were the same as those described above.

### Immunofluorescence Analysis and Hematoxylin-Eosin (HE) Staining

At 48 h after cerebral ischemia, paraffin-embedded segments were used to evaluate the expression of GFAP, LCN2, p-STAT3, and p-JAK2 according to immunohistochemical protocols (*n* = 5 per group). Brain tissues were fixed in 4% paraformaldehyde in PBS (0.01 M, pH 7.4) for 24 h at 4°C, dehydrated in a graded series of alcohols, and embedded in paraffin. Brain tissues were cut into 5-μm-thick sections using a Leica^®^ RM1850 rotary microtome (Leica Microsystems, Germany). To eradicate endogenous peroxidase activity, the sections were incubated in 3% H_2_O_2_ and 3% normal goat serum. The sections were then incubated for 1 h at 37°C using the following rabbit polyclonal antibodies: LCN2 (Abcam, 1:200), GFAP (Proteintech, 1:200), p-JAK2 (Tyr1007/1008) (Abcam, 1:50), JAK2 (Abcam, 1:200), p-STAT3 (Tyr705) (Cell Signaling Technology, 1:100), and STAT3 (Cell Signaling Technology, 1:200). The sections were rinsed with PBS and incubated with secondary antibodies (Cell Signaling Technology, 1:100) at room temperature for 45 min; then, the sections were developed by diaminobenzidine and counterstained with hematoxylin. Secondary antibodies, biotinylated conjugates, and diaminobenzidine were obtained using a streptavidin-peroxidase kit, and the resulting signals were imaged. Brain sections were counterstained with Hoechst 33342 (10 μg/mL) for 15 min to distinguish the nucleus. To obtain color images, a 20 × laser scanning confocal microscope (Olympus FV1200, Tokyo, Japan) was utilized.

### Determination of Chemokine/Cytokine Expression in Brain Extracts, Serum Samples and Astrocytes

The chemokine and cytokine concentrations in brain hemisphere extracts, serum samples and astrocytes conditioned in medium were quantified by the EMD Millipore’s MILLIPLEX^®^ MAP Rat Cytokine/Chemokine Magnetic Bead assay in compliance with the manufacturer’s instructions. The chemokine and cytokine concentrations from tissues containing ischemic penumbra were harvested at 48 h after cerebral ischemia was extracted by EMD Millipore’s buffer. Blood serum was collected using the abdominal aorta method. The chemokine and cytokine concentrations in astrocytes conditioned in medium were harvested after OGD induction. The cytokines TNFα, IL-1α, IL-1β, and IL-6 and the chemokine CXCL10 (IP-10) were analyzed. The median fluorescence intensity was assayed on a FLEXMAP 3D^TM^ system. A five-parameter logistic method was performed to approximate the cytokine and chemokine concentrations in both the brain homogenates and serums samples.

### Statistical Analyses

All statistical data were analyzed using GraphPad Prism software (San Diego, CA, United States). The results are expressed as the means ± SEM. Each experiment was performed in triplicate. Multiple group comparisons were analyzed using one-way analysis of variance (ANOVA). Student’s *t*-test was conducted to analyze intergroup comparisons. For neurological studies, two-way ANOVA was conducted for intergroup pairwise comparisons as a function of time. *P*-values of (*P* < 0.05) were considered statistically significant.

## Results

### EGB Protected Against Cerebral Ischemia-Induced Brain Injury

**FIGURE 1:**
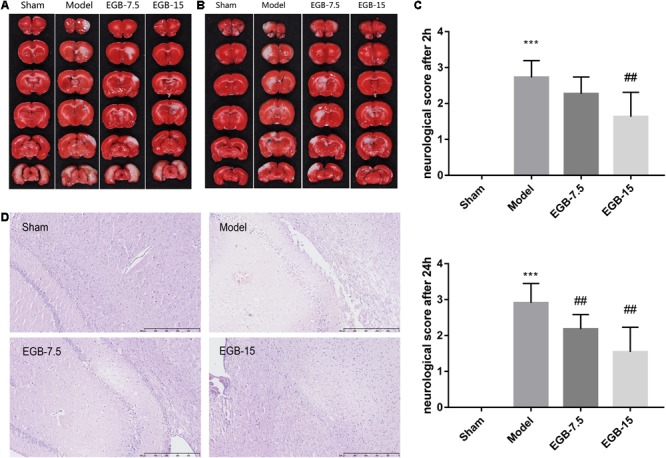
EGB treatment reduced ischemic infarct volume in the cerebral ischemia model. **(A,B)** Cerebral infarct volume was assessed via TTC staining 48 h after cerebral ischemia. Neurological score **(C)** of rats after cerebral ischemia were assessed using a five-point scale system. Data are expressed as mean ± SEM (*n* = 10). ^∗∗∗^*p* < 0.01 vs. sham group; ^##^*p* < 0.01 vs. the model groups. The ischemic penumbra area in the box **(D)** was assessed for neuronal apoptosis using HE staining.

*Ginkgo biloba* extract was injected into the duodenum of rats after the occurrence of cerebral ischemia. Brain infarct volumes were determined by TTC assay 48 h after surgery. EGB dose-dependently reduced brain infarct volume. Accordingly, the neurological deficit scores mirrored the results of infarct volume, indicating the protective effects of EGB. These data suggested a neuroprotective role of EGB in reperfusion after ischemia. Compared to the cerebral ischemia group, the EGB-7.5 (7.5 mg/kg, daily) and EGB-15 (15 mg/kg, daily) groups had a significant reduction in neurological deficit scores (*P* < 0.05). Moreover, the neurological deficit score in the EGB-15 group was less than that in the EGB-7.5 group. **Figure 1C** shows the neurological deficit scores for all groups at 2 and 24 h after surgery and the occurrence of cerebral ischemia, respectively.

**Figures 1A,B** show the infarct volume of all groups at 48 h after cerebral ischemia occurred. In the cerebral ischemia group, extensive lesions were observed in the striatum, hippocampus, and cortex. Consistent with the neurological deficit scores, the group treated with EGB (15 mg/kg, daily) had reduced infarct volume and showed a significant therapeutic effect compared with the cerebral ischemia group.

Hematoxylin-Eosin staining was observed to identify the histological changes of brain neurons for all groups (**Figure 1D**). Cortex sections and hippocampal regions stained with HE presented with neuronal loss and signs of cerebral edema, and swollen cells were observed in the ipsilateral frontal cortex. After surgery, several apoptotic neurons were observed with karyopyknosis, cell gaps and debris. EGB (15 mg/kg, daily) significantly alleviated the symptoms of apoptosis in a dose-dependent manner. Therefore, these results indicated that 15 mg/kg EGB can reduce brain injury caused by cerebral ischemia.

### Ultrastructural Changes Associated With EGB Treatment

**FIGURE 2:**
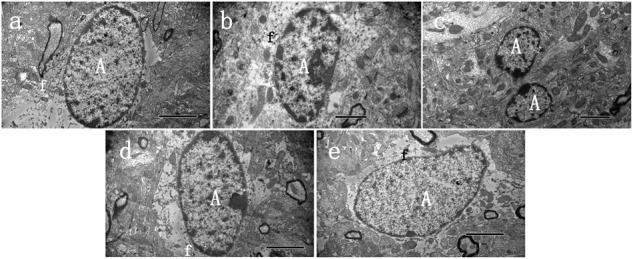
Ultrastructural characteristics of astrocytes 48 h after cerebral ischemia. Representative transmission electron microscopy images of astrocytes in the peri-infarct area of stroke, at 48 h after cerebral ischemia, in three groups of animals. **(A)** Normal astrocyte was observed in sham group, **(B)** Swollen activated perineuronal and perivascular astrocytes were seen in model groups, which demonstrated activation as evidenced by a significant amount of chromatin condensation atop the nuclear membrane and large nucleolus. The astrocytes were characterized by dispersed residual organelles and swollen mitochondria. Astrocyte fusion was often observed **(C)**. **(D,E)** Less damage was observed in the EGB treatment group. A astrocyte, f—fibrils. Bars: **A,C,D,E** 2 μm; **B** 1 μm.

Electron microscopy studies were performed to generate a detailed evaluation of astrocyte phenotypes in the peri-infarct area following a stroke (**Figure 2**). The cytoplasm of the astrocytes was occasionally observed to be completely empty, with dispersed organelles and fibrils (**Figures 2A–E**). In the model group, swollen astrocytes adhered to the damaged area, which indicated activation, as evidenced by a significant amount of chromatin condensation on top of the nuclear membrane and a large nucleolus. Activated astrocytes and two-cell fusion were also observed (**Figure 2C**). These astrocytes were not observed in the control group. Finally, EGB treatment (15 mg/kg, daily) reduced the damage to brain tissue elements in a dose-dependent manner, and this tissue had a more viable appearance.

### Effect of EGB on Inflammatory Markers

**FIGURE 3:**
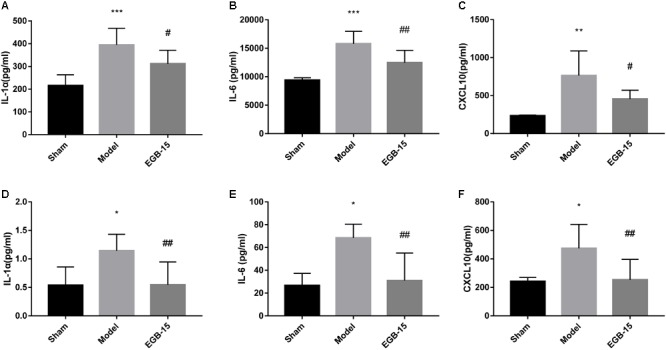
Effects of EGB treatment on the level of cytokines/chemokines in the brain after cerebral ischemia. **(A–F)** Analysis showing the relative levels of the pro-inflammatory mediators IL-1α, IL-6, and CXCL 10 (IP-10), **(A–C)** was in brain, **(D–F)** was in serum. Data are expressed as mean ± SEM (*n* = 5). ^∗^*p* < 0.05, ^∗∗^*p* < 0.01 and ^∗∗∗^*p* < 0.001 vs. sham group; ^#^*p* < 0.05 and ^##^*p* < 0.01 vs. the model groups.

A significant difference in cytokine levels was observed after surgery. For example, at 48 h, pro-inflammatory IL-1α, IL-1β and chemokine CXCL10 (IP-10) were noted in the ipsilateral hemisphere of the model group but were not observed in the control group. The degree of statistical significance was calculated at *p* < 0.001 for IL-1α, *p* < 0.001 for IL-1β, and *p* < 0.01 for the chemokine CXCL10. However, EGB significantly reduced these cytokine levels compared with those of the cerebral ischemia group (*p* < 0.05, EGB-15 group vs. model group; *p* < 0.01, EGB-15 group vs. model group) (**Figures 3A–C**). Additionally, identical results were found in the rats’ serum. The levels of IL-1α, IL-1β and CXCL10 in the model group were markedly higher than those of the sham group (*p* < 0.05) (**Figures 3D–F**). Similarly, the EGB-15 group presented with reduced cytokine levels compared with the cerebral ischemia group (*p* < 0.01, EGB-15 group vs. model group).

### EGB Alleviates LCN2 Up-Regulation in a Rodent Model of Cerebral Ischemia

**FIGURE 4:**
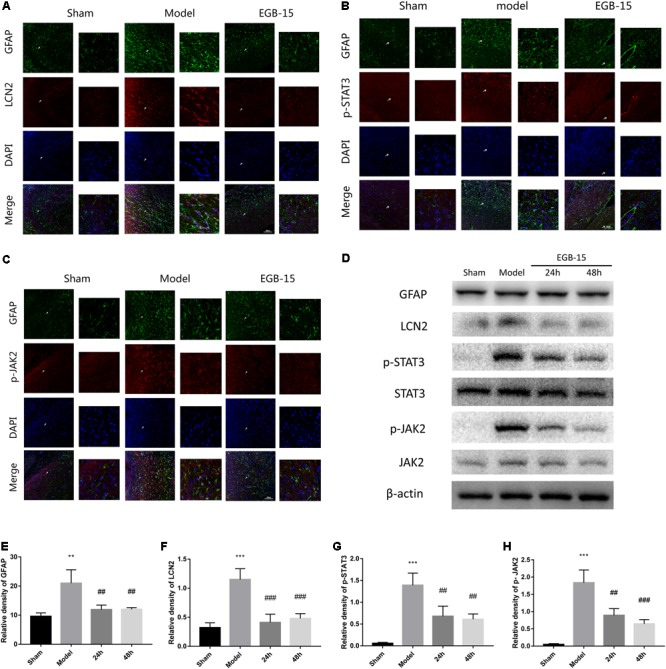
Effects of EGB on the activation of astrocytes and the expression of LCN2, p-JAK2, p-STAT3, in cerebral ischemia rats. **(A–C)** Double-immunofluorescence staining for astrocytic LCN2, p-STAT3, p-JAK2, and GFAP expression in the ischemic penumbra area after cerebral ischemia. The cells indicated with an arrow were magnified. Scale bar = 20 μm. **(D–H)** Western blots and quantitative analysis of GFAP, LCN2, p-JAK2, and p-STAT3 expression are expressed as mean ± SEM (*n* = 4). ^∗^*p* < 0.05, ^∗∗^*p* < 0.01 and ^∗∗∗^*p* < 0.001 vs. sham group; ^#^*p* < 0.05, ^##^*p* < 0.01 and ^###^*p* < 0.001 vs. model groups.

Immunofluorescence staining and Western blot analysis revealed a significant increase in brain LCN2 protein levels after cerebral ischemia. An EGB injection into the duodenum reduced cerebral ischemia-induced LCN2 levels. To determine the cellular localization of LCN2 protein in the hippocampus of cerebral ischemic rats, double-immunofluorescence staining for LCN2 and GFAP was performed in brain sections after cerebral ischemia. LCN2 protein expression was localized to within GFAP-positive astrocytes. Consistent with the results shown in **Figure 4**, the expression of LCN2 was induced, and the number of astrocytes in the cortex was increased in rats with cerebral ischemia compared with the sham-operated rats. However, EGB significantly reduced GFAP levels. The effects of EGB on astrocyte activation, LCN2, p-STAT3, and p-JAK2 expression in the ipsilateral peri-infarct cortical area (-1.7 to -1.9 mm from the bregma) following cerebral ischemia were captured with a laser confocal microscope (Olympus FV1200, Japan). To determine the potential role of EGB and LCN2 on neuroinflammation caused by ischemic injury, astrocyte activation status was assessed. EGB treatment (15 mg/kg) suppressed the activation of astrocytes in the ischemic hemisphere, as suggested by the reduced GFAP levels detected by immunofluorescence staining and Western blotting (*p* < 0.05, EGB group vs. model group). In addition, double-immunofluorescence staining also demonstrated that astrocytic p-STAT3 and p-JAK2 levels were markedly increased after cerebral ischemia but were diminished by EGB treatment (**Figures 4A–C**). The expression of GFAP, LCN2, p-STAT3, and p-JAK2 was determined using Western blot (**Figures 4D–H**). LCN2 was observed to increase in the cerebral ischemia group compared to the sham group (*p* < 0.05). Finally, EGB markedly downregulated LCN2 expression in the cerebral ischemia group compared to the sham group (*p* < 0.01). A similar pattern of expression was noted for p-STAT3 and p-JAK2.

### EGB Attenuates OGD-Induced Injury in Astrocytes

CCK8 assays revealed that cell viability was significantly reduced by OGD-induced injury. EGB (50∼200 mg/L) was observed to have a dose-dependent toxic effect on the viability (CCK8 assay) of the human astrocytes. However, treatment with EGB (12.5∼50 mg/L) attenuate the OGD-induced decrease in cellular activity. Finally, the pesticide effect was noted in the 2.5 mg/L and 5 mg/L EGB groups (*p* < 0.05, vs. OGD group); therefore, 5 mg/L EGB was administered in the following *in vitro* tests (Supplementary Figure S1).

**FIGURE 5:**
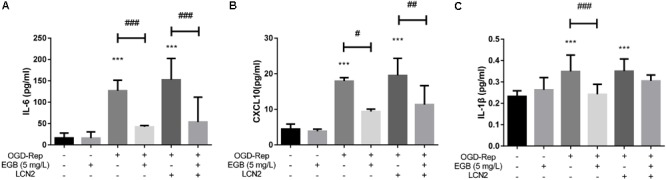
EGB suppressed OGD-induced inflammation in astrocytes *in vitro*. **(A–C)** Analysis showing the relative levels of the pro-inflammatory mediators IL-1β, IL-6, and CXCL10 (IP-10). Data are expressed as mean ± SEM (*n* = 5). ^∗^*p* < 0.05, ^∗∗^*p* < 0.01 and ^∗∗∗^*p* < 0.001 vs. control group; ^#^*p* < 0.05, ^##^*p* < 0.01 and ^###^*p* < 0.001 and vs. the indicated groups.

To determine the role of LCN2 in the anti-inflammatory effects of EGB, LCN2 was overexpressed in human astrocytes. The cells were incubated under OGD settings to simulate an ischemic model *in vitro*. After OGD induction, the EGB treatment group had significantly reduced levels of the pro-inflammatory cytokines IL-6, IL-1β, and CXCL10 compared to the model group (*p* < 0.05). This effect was enhanced by LCN2 overexpression (*p* < 0.05, vs. control group) (**Figures 5A–C**).

**FIGURE 6:**
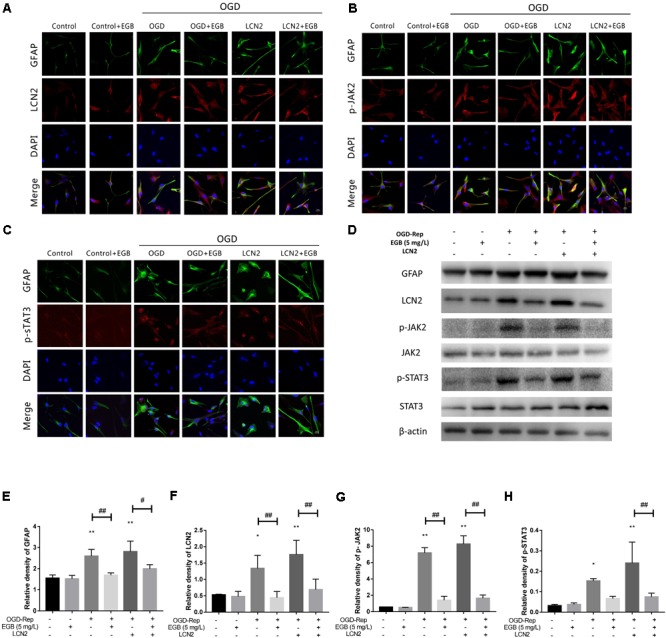
The effects of EGB on the activation of astrocytes and the expression of astrocytic LCN2, p-JAK2, and p-STAT3 after OGD induction *in vitro*. **(A–H)** Double-immunofluorescence staining for GFAP, LCN2, p-JAK2, and p-STAT3 in astrocytes after OGD induction. Scale bar = 20 μm. Western blots and quantitative analysis of GFAP, LCN2, p-JAK2, and p-STAT3 expression. Data are expressed as mean ± SEM (*n* = 3). ^∗^*p* < 0.05 and ^∗∗^*p* < 0.01 vs. control group; ^#^*p* < 0.05 and ^##^*p* < 0.01 vs. the indicated groups.

A change in astrocyte morphology was determined by GFAP staining (**Figures 6A–C**). OGD-induced astrocyte activation was confirmed by lengthened cellular protuberances, and this effect was enhanced by LCN2 overexpression. EGB alleviated the activation of astrocytes after OGD induction. To investigate the downstream anti-inflammatory targets of EGB, the expression levels of LCN2, p-JAK2, and p-STAT3 were examined in human astrocytes. Compared to the control group, the OGD-stimulated model group presented with dramatically increased levels of GFAP, LCN2, p-JAK2, and p-STAT3 (*p* < 0.05, *p* < 0.01) (**Figures 6A–H**). These effects were enhanced by LCN2 overexpression, as evidenced by immunofluorescence staining and Western blotting (**Figure 6**). EGB was observed to markedly reduce GFAP, LCN2, p-JAK2, and p-STAT3 levels after OGD stimulation (*p* < 0.05, *p* < 0.01). Interestingly, in human astrocytes, EGB significantly decreased the level of LCN2 overexpression and suppressed the phosphorylation of STAT3 and JAK2 after OGD. No differences between the control group and the EGB-treated control group were observed.

## Discussion

In this study, a novel signaling pathway involving LCN2 for the EGB-mediated dysfunction of reactive astrocyte properties in ischemia was reported. EGB markedly reduced stroke damage and protected the brain cortex from damage by decreasing infarct volume in cerebral ischemic rats. EGB ameliorated the activation of astrocytes, reduced the phosphorylation of STAT3 and JAK2 and decreased LCN2 expression *in vivo* and *in vitro*. However, LCN2 overexpression was observed to markedly reverse this result. Therefore, these results suggested that EGB exerts potent therapeutic properties in cerebral ischemia by targeting astrocytic LCN2 and suppressing neuro-inflammatory injury via the JAK2/STAT3 pathway.

Inflammation has long been known to influence the brain after cerebral ischemia, and modulating adaptive immunity may prevent the post-ischemic immune response from incurring tissue damage. Inflammatory signaling is instrumental in all stages of the ischemic cascade, from the early damaging events triggered by arterial occlusion, to the late regenerative processes underlying post-ischemic tissue repair (Iadecola and Anrather, 2011). Since 1964, the effect of EGB on several disorders and diseases has been examined. Specifically, the investigated effects included (but were not limited to) cerebral insufficiency, Alzheimer’s disease, POAD, and thrombosis (Maclennan et al., 2002). It has been reported that EGB, as a multifunctional neuroprotective agent, can reverse the brain oxidative damage induced by hydrogen peroxide and amyloid β-peptide, and also can suppress activation of astrocytes and microglia (Brunetti et al., 2006; Tulsulkar and Shah, 2013). In the present study, EGB was observed to reduce cerebral infarction and neuronal apoptosis in the ischemic hemisphere. Neurological disorders were also ameliorated in cerebral ischemic rats treated with EGB. Accordingly, this result provided the first evidence that EGB diminishes ischemic brain injury. The inflammatory response has both advantages and disadvantages (Fann et al., 2013), as it originally contributes to ischemic brain injury but then assists tissue regeneration (Chamorro and Hallenbeck, 2006).

The inflammatory response is characterized by a release of pro-inflammatory cytokines, including TNF-α, IL-1α, IL-6, and IL-18, and chemokines, including CCL2, CXCL10, and CXCL12 (Ambrosini and Aloisi, 2004). This release is mediated by neurons, astrocytes, microglia, and endothelial cells and can cause neuronal and glial cell death during cerebral ischemia (Allan and Rothwell, 2001). Indeed, it is reasonable to assume that modulating the function of pro-inflammatory cytokines and chemokines in strokes significantly affects infarct evolution (Lambertsen et al., 2012). Here, we demonstrated that EGB markedly reduced pro-inflammatory cytokines/chemokines levels, including those of IL-6, IL-α and CXCL10, in tissues surrounding the cerebral infarction. Additionally, EGB decreased these pro-inflammatory cytokines and chemokines in the serum.

Astrogliosis has the potential to lead to harmful effects, namely, neuroinflammation, and to interfere with synapse sprouting (Silver and Miller, 2004; Sofroniew and Vinters, 2010). Research has shown that cytokines and inflammatory responses can mediate astrocyte gene expression and physiology by impacting synaptic and neuronal functions (Sofroniew, 2014). However, the lack of effective therapeutic treatment targeting astrogliosis is becoming an issue of concern for clinical treatment. LCN2 is a member of the secreted lipocalin protein family. To date, several studies have confirmed the detrimental role of LCN2 in ischemic brains (Jin et al., 2014; Suk, 2016). In addition, several clinical trials support the experimental laboratory findings that LCN2 is a crucial component of neuroinflammation that mediates brain injury in cerebral ischemia and other pathological conditions (Chan et al., 2012; Fernandez-Cadenas et al., 2013). A recent study showed that LCN2 is a reactive astrocyte marker and an autocrine mediator of reactive astrogliosis (Lee et al., 2009). LCN2 amplifies neurotoxic inflammation through the activation of microglia/astrocytes and the induction of pro-inflammatory cytokines and chemokines like IL-6 and CXCL10 (Hamzic et al., 2013; Xing et al., 2014). These results suggest EGB as a new therapeutic target for suppressing innate immune responses during stroke. As demonstrated in the present study, marked astrocyte activation and LCN2 up-regulation were observed throughout the ischemic cortex area after cerebral ischemia, while EGB treatment significantly inhibited the activation of astrocytes and markedly decreased LCN2 expression. Astrocytes were also responsive to OGD-induced inflammation, as evidenced by LCN2 up-regulation. Therefore, these results provide evidence for the suppression of astrocytic LCN2 and for its contribution to the anti-inflammatory effects of EGB after cerebral ischemia occurs.

Constitutive activation of the JAK-STAT signaling pathway has been reported in cerebral ischemia. Recent investigations support a role for JAK-STAT signaling in hematopoiesis (Kisseleva et al., 2002), immune responses (Ivashkiv, 2000), cellular homeostasis (Schindler, 2002), gliogenesis (Sauvageot and Stiles, 2002), and reactive astrogliosis (Na et al., 2007; Herrmann et al., 2008). Furthermore, the activation of STAT3 by phosphorylation at Tyr705 is increased after CNS insults (Sriram et al., 2004; Yamauchi et al., 2006). STAT3 activation has been associated with increased neuroinflammation in cerebral ischemia (McGuckin et al., 2013), of which found that the JAK2-STAT3 pathway was involved in the LCN2 induction of CXCL10 secretion and possibly other phenotypic changes associated with reactive astrogliosis (Lee et al., 2011). In the present study, astrocytic p-STAT3 and p-JAK2 were up-regulated after cerebral ischemia *in vivo* and after OGD *in vitro*. However, EGB acted as an effective JAK-STAT inhibitor in the cerebral ischemia model and in OGD-induced astrocytes by inducing anti-inflammatory alterations. In addition, LCN2 overexpression increased both p-STAT3 and p-JAK2 expression, but this effect was reversed by EGB treatment. These results confirmed that the JAK2/STAT3 signaling pathway is involved in LCN2-induced GFAP expression and plays a key role in LCN2 activity in astrocytes (Lee et al., 2011). Therefore, LCN2-JAK2/STAT3 signaling may mediate the neuroprotective effect of EGB. Thus, we inferred that EGB suppressed neuroinflammation by inhibiting the phosphorylation of STAT3 and JAK2 in astrocytes via LCN2 suppression. However, these results do not explain all mechanisms underlying the protective effects of EGB. The alleviation of LCN2 expression was postulated as a key mechanism, as some potential indirect neuroprotective effects of EGB may still exist.

## Conclusion

To conclude, EGB has been demonstrated to mediate neuroinflammation, which protects against ischemic brain injury by inhibiting astrogliosis. JAK2/STAT3-signaling regulation of LCN2 expression may be involved in astrogliosis and neuroinflammation. Lastly, these results imply that LCN2 could be used as a target to therapeutically modulate astrocytic responses and as an inflammatory tool for exploring the possibility of EGB as a therapeutic agent.

## Availability of Data and Materials

All the datasets and materials supporting the conclusions of this article are provided in the manuscript, which includes the article and the additional files.

## Author Contributions

YeZ performed the research study. YoZ and JL designed the research study. BY and MY contributed to data analysis. MS, LX, CL, DC, and FT contributed to the drafting and revising of the manuscript and accepted the final manuscript.

## Conflict of Interest Statement

The authors declare that the research was conducted in the absence of any commercial or financial relationships that could be construed as a potential conflict of interest.

## Abbreviations

CCK8: cell counting kit-8
CNS: central nervous system
CXCL: chemokine (C-X-C motif) ligand
EGB: *Ginkgo biloba* extract
FBS: fetal bovine serum
GFAP: glial fibrillary acidic protein
HE: hematoxylin-eosin
HPLC: high performance liquid chromatography
IL-1α: interleukin-1α
IL-1β: interleukin-1β
IL-6: interleukin -6
JAK2: Janus kinase 2
LCN2: Lipocalin-2
OGD: oxygen glucose deprivation
POAD: peripheral occlusive arterial disease
STAT3: signal transducer and activator of transcription 3
TNF-α: tumor necrosis factor-alpha
TTC: triphenyltetrazolium chloride
UV: ultraviolet
