# Supplementary material for: Ginkgo biloba Extract Inhibits Astrocytic Lipocalin-2 Expression and Alleviates Neuroinflammatory Injury via the JAK2/STAT3 Pathway After Ischemic Brain Stroke
Source: Front Pharmacol. 2018 May 16;9:518. doi: 10.3389/fphar.2018.00518 (PMC5964562; doi:10.3389/fphar.2018.00518)
Supplement: Supplementary file 1 [file Image_1.pdf]

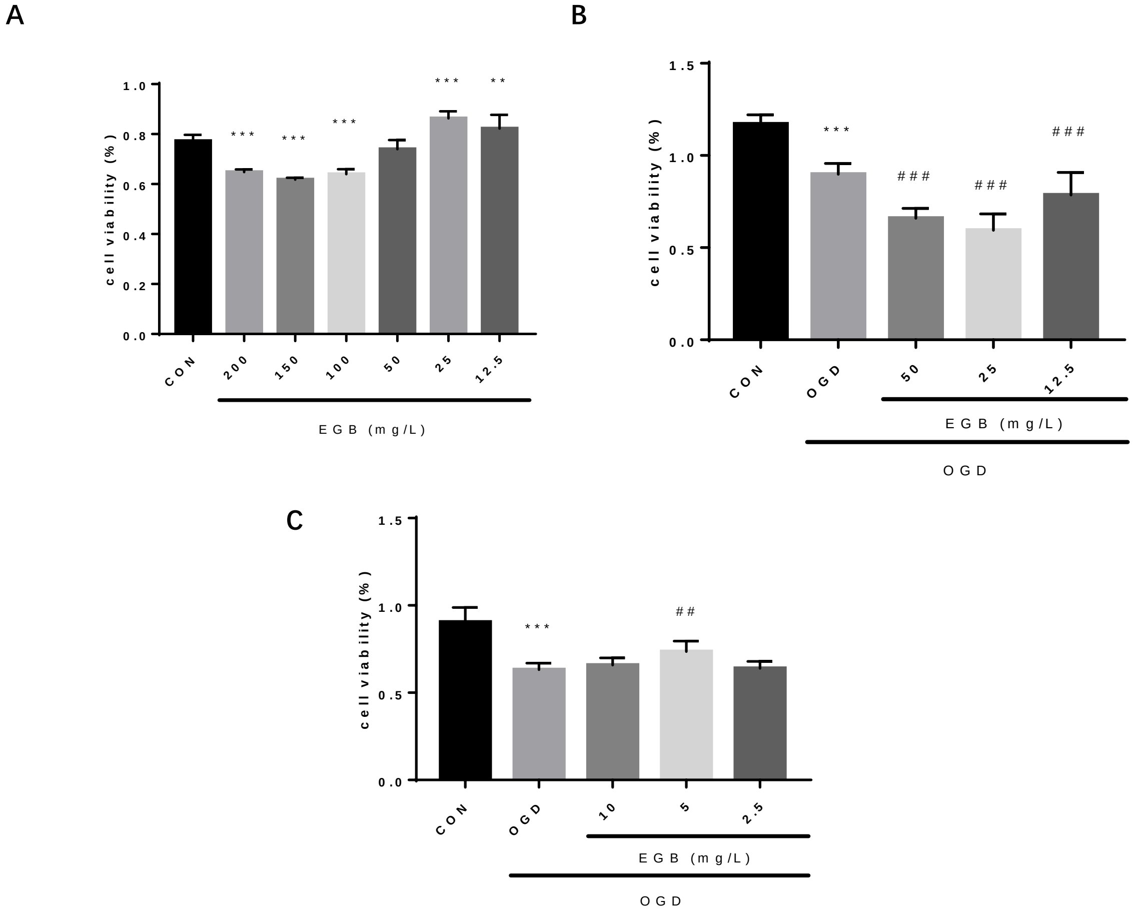

Figure S1. The effect of EGB on cell viability in astrocytes.

A. MTT assay detecting the cell viability treated with different dosages of EGB for 24 h.

B-C MTT assay detecting the cell viability treated with different dosages of EGB for 6h during OGD induction. Data are expressed as mean ± SEM (n = 6). $*P < 0.05$, $**P < 0.01$, $** P < 0.001$ *vs.* the control groups, $^{\#}P < 0.05$, $^{\#\#}P < 0.05$ and $^{\#\#\#}P < 0.001$ *vs.* the OGD groups.
